# Heterogeneous effects of socio-economic and cultural factors on fertility differentials in Burundi and Morocco during their fertility transition periods: a retrospective, cross-sectional and comparative study

**DOI:** 10.11604/pamj.2023.45.161.36150

**Published:** 2023-08-15

**Authors:** Jean Claude Nibaruta, Bella Kamana, Mohamed Chahboune, Milouda Chebabe, Saad Elmadani, Jack Edward Turman, Morad Guennouni, Hakima Amor, Abdellatif Baali, Noureddine Elkhoudri

**Affiliations:** 1 Hassan First University of Settat, Higher Institute of Health Sciences, Laboratory of Health Sciences and Technologies, Settat, Morocco,; 2 Hassan II University, Ibn Rochd University Hospital of Casablanca, Medical Biology Laboratory, Casablanca, Morocco,; 3 Indiana University, Richard M. Fairbanks School of Public Health, Department of Social and Behavioral Sciences, Indianapolis, Indiana, USA,; 4 Chouaîb Doukkali University, Higher School of Education and Training, Science and Technology Team, El Jadida, Morocco,; 5 Cadi Ayyad University of Marrakech, Semlalia Faculty of Science, Department of Biology, Marrakech, Morocco

**Keywords:** Fertility transition, children ever born (CEB), socioeconomic factors, cultural factors, demographic and health surveys, Burundi, Morocco

## Abstract

**Introduction:**

few studies have examined the factors influencing fertility differentials and the variation in their effects in countries with different socioeconomic and cultural backgrounds and different fertility transition paces. To address this gap, our study sought to first identify the factors that influenced fertility differentials in Morocco and Burundi during their fertility transition periods, and then to compare the effects of these factors between the two countries.

**Methods:**

using data from the 2003-4 Morocco and 2010 Burundi Demographic and Health Surveys, bivariable and multivariable Poisson regression analyses offset by the natural logarithm of the women´s age were performed to identify the socioeconomic and cultural factors that influenced fertility differentials in Morocco and Burundi during their fertility transition.

**Results:**

our main findings showed that the total number of children ever born ranged from 0 to 17 with a mean of 2.71 ± 2.89 in Burundi and from 0 to 16 with a mean of 1.88 ± 2.80 in Morocco. In Burundi, both socioeconomic and cultural factors like rural residence adjusted incident rate ratio (AIRR) = 1.159, 95% CI: 1.103 - 1.217, P=0.020), women´s illiteracy (AIRR=1.465, 95% CI: 1.241- 1.729, P <0.001) and agricultural profession (AIRR=1. 332, 95% CI: 1.263 - 1.401, P = 0.004), household poverty (AIRR= 1.381, 95% CI: 1.223 - 1.431, p<0.001), infant mortality (AIRR= 1.602, 95% CI: 1.562 - 1.643, p<0.001), early marriage (AIRR= 1.313, 95% CI: 1.264 - 1.364, p<0.001), lack of knowledge of any contraceptives (AIRR= 1.263, 95% CI: 1.125 - 1.310, p = 0.003) and failure to use modern contraceptives (AIRR= 1.520, 95% CI: 1.487 - 1.611, p<0.001) were associated with high number of children ever born. However, in Morocco socioeconomic factors like residence place, women´s agricultural profession and household poverty were not significant. In this country, women´s illiteracy (AIRR=1.428, 95% CI: 1.315 - 1.551, P <0.001), lack of access to mass media (AIRR= 1.241, 95% CI: 1.108 - 1.375, p = 0.006), infant mortality (AIRR=1.222, 95%CI: 1.184 - 1.361, p<0.001), early marriage (AIRR1.481, 95% CI: 1.435 - 1.529, p<0.001), lack of knowledge of any contraceptives (AIRR1.508, 95% CI: 1.409 - 1.613, p<0.001) and failure to use modern contraceptives (AIRR1.745, 95% CI: 1.627 - 1.863, p<0.001) were associated with high fertility but with different effects than in Burundi.

**Conclusion:**

the evidence from this study suggests that interventions to accelerate the fertility transition processes in Burundi and many other countries with slow fertility transitions should be designed and implemented according to each country's local context.

## Introduction

**Figure 1:**
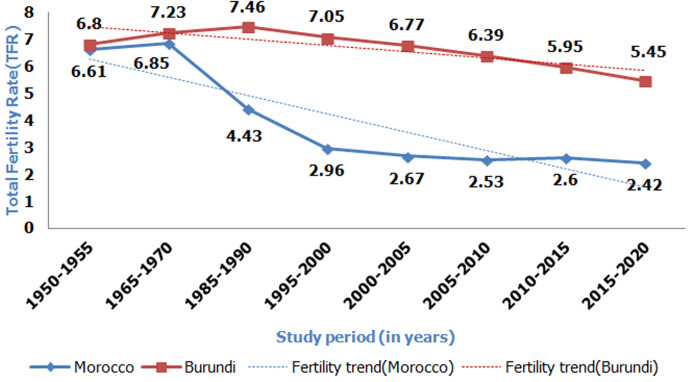
fertility trends in Morocco and Burundi from 1950 to 2020

Fertility transition is characterized by enormous disparities between regions and within countries. While rapid fertility declines were observed in Asia, Latin America, Europe and Northern Africa, sub-Saharan African (SSA) countries are experiencing late and very slow fertility transitions [1,2]. Morocco is one of the North African countries where fertility decline is more remarkable in terms of speed and magnitude over the past 50 years [3]. The fertility transition is most often characterized by a decline in the total fertility rate (TFR), an indicator defined as the mean number of children a woman would have if she survives all her childbearing years by giving birth according to a current schedule of age-specific fertility rates [4]. Thus, analysis of Figure 1, made using data from the United Nations [5], reveals that Morocco was close to natural fertility in 1950 -1955 with a TFR of 6.6. From 1965 to 1970, the TFR increased, reaching a peak of 6.85. The TFR decreased significantly from 6.85 to 2.96 in 1970 - 2000, a decrease of about 4 children per woman in 30 years, and from 2.96 to 2.42 in 2000 - 2020. Unlike Morocco, Burundi is one of the SSA countries that started the fertility transition process late and is still experiencing a very slow pace of fertility decline [6]. Figure 1shows that until the early 1990s, Burundi had not yet begun the fertility transition, whereas Morocco had already initiated it 20 years earlier. Therefore, the TFR was estimated at 7.46 in Burundi compared to 4.43 in Morocco. The TFR increased from 6.8 in 1950 - 1955 to 7.23 in 1965 -1970, reaching a peak of 7.46 in 1985 - 1990. A progressive decline in fertility began in the 1990s, but at a very slow pace compared to that of Morocco.

Several studies asserted that fertility differentials are influenced by socioeconomic [7-9], cultural [10-12], biological [13], and contextual [14] factors and the effects of these factors could vary between and within countries depending on the local context of each country [9]. Thus, the strong fertility differentials observed between Morocco and Burundi during their fertility transition periods could be explained by possible variation in the effects of these factors, given that the Moroccan and Burundian socioeconomic and cultural contexts remain very different. Indeed, Morocco was already among the most urbanized countries in North Africa during its fertility transition period as its urbanization rate was 54.6% in 2004 [15], in addition to a mechanized agricultural sector [16] and a significant improvement in the households´ socio-economic conditions especially in urban areas [3,17]. There were already notable successes in promoting maternal health and child survival [18]. The great success of the national Family Planning(FP)program in Morocco [3] would have reduced socioeconomic gaps in access to FP services [19], and significantly increased awareness and use of modern contraceptive methods [3,17]. As an almost entirely Muslim country, Morocco recognizes marriage as the only framework for childbearing and this may have contributed to the decline in fertility [3]. Unlike Morocco, Burundi remains among the poorest countries in the world [20] and the least urbanized with an urbanization rate of 10.6% in 2010 [15]. The agricultural sector remains traditional and therefore encourages most Burundian couples to give birth to many children given their important role in their parents' farming activities [21]. Moreover, maternal and child mortality rates are still high despite significant improvements [21,22]. Significant urban and rural gaps in knowledge of any contraceptive method [21], unmet need for family planning [23], and use of modern contraceptives [24] persist in Burundi. Unlike Morocco, Burundi is a predominantly Christian country [22]. Therefore, marriage is also the only recognized framework in which childbearing is permitted. However, other illegal childbearing settings such as cohabitation [22] and unwanted teenage pregnancies [25] are still common.

However, many studies have focused on analyzing the factors that influenced fertility differentials in countries that have almost the same socioeconomic and cultural characteristics and therefore almost the same fertility transition paces [3,9,26,27]. Few studies [28] have examined the factors influencing fertility differentials and the variation in their effects in countries with different socioeconomic and cultural backgrounds and different fertility transition paces. To address this gap, our study sought to first identify the socioeconomic and cultural factors that influenced fertility differentials in Morocco and Burundi during their fertility transition periods and then to compare the effects of these factors between the two countries. We expect that the results of this study could contribute to a better understanding of the extent to which Burundi and many other countries with very slow fertility transitions could accelerate them using/not the same interventions as those used by other countries, like Morocco, that succeeded in accelerating their fertility transitions.

## Methods

**Study design and setting:** this is a retrospective, cross-sectional and comparative study using data from the Demographic and Health Surveys (DHS) conducted from October 15^th^, 2003 to February 2915^th^, 2004 in Morocco (2003-04 Morocco DHS) [18] and from August 2915^th^, 2010 to January 3015^th^, 2011 in Burundi (2010 Burundi DHS) [6]. The 2003-04 Morocco DHS and 2010 Burundi DHS are nationally representative surveys with a cross-sectional design conducted as part of the DHS program [29] to generate national-level updated estimates of basic demographic and health indicators [6,18]. We used the two surveys to identify the socioeconomic and cultural factors that influenced fertility differentials in Morocco and Burundi during their fertility transition periods, because both Morocco and Burundi were in the midst of fertility transitions during 2003-04 [18] and 2010-11 [6] periods respectively.

**Participants:** participants in both surveys were women of reproductive age (women aged 15-49).

**Eligibility criteria:** any woman aged 15-49 who usually resided in the selected households or a female visitor who spent the night before the day of the interview in the selected household [6,18], and women who gave informed consent to participate in the survey.

**Exclusion criteria:** any woman under 15 or over 49 years old and woman´s refusal to participate in the survey.

**Sample size and sampling method:** this study used two samples of 9,389 and 1,769 women aged 15-49 years for the 2010 Burundi and 2003-04 Morocco DHS surveys, respectively. A two-stage cluster sampling procedure was used to obtain the two samples [6,18].

**Bias control:** to minimize bias, a pilot survey was conducted to adapt the data collection tools and data collection was carried out by specifically trained interviewers. In addition, in each team of interviewers, a supervisor was always in charge of controlling the quality of the data collected [6,18].

### Study variables

**Outcome variable:** this study used the total number of Children Ever Born (CEB) as the outcome variable. The total number of CEB is a measure of each woman's fertility level up to the time the 2003-4 Morocco and 2010 Burundi DHS survey data were collected. The total number of CEB was used as the dependent variable because the total number of CEB of women in the 15 to 49-year-old cohort captured both current and past fertility behavior [30]. In addition, as this study plans to use a Poisson regression model that is typically used when the dependent variable is a count variable such as the number of CEB [21,30], we considered that the number of CEB would be the most appropriate outcome variable in this study.

**Independent variables:** using a preliminary literature review, socioeconomic and cultural variables were selected from the two databases to analyze their potential association with fertility differentials in the both countries. Selected variables include women´s age ( women´s current age divided into 7 classes of 5 years of interval), place of residence (women´s type of place of residence: urban or rural), marital status (women´s current marital status), women and husband's education (women/husband´s highest educational level), women/husband´s profession (women/husband´s current activity), wealth quintile (household wealth index), mass media access (variable obtained by combining variables like frequencies of listening to radio, watching TV and reading newspapers), experience of infant mortality (whether the women has/ not already lost at least one child before his/her fifth birthday), age at first marriage (age at first marriage /cohabitation), knowledge of any contraceptive methods (whether a women has/not knowledge about any contraceptive methods) and use of modern contraceptives (current use of modern contraceptive methods).

**Statistical analysis:** the statistical software STATA version 14.2 was used during statistical analyses. Since the 2003-4 Morocco and 2010 Burundi DHS data were obtained using a two-stage stratified cluster sampling process, the data were first weighted using the STATA svyset command to obtain unbiased parameter estimates and standard errors [31]. Then, frequencies and percentages (for categorical variables), means and standard deviations (for quantitative variables) were computed to describe the socio-demographic characteristics of the two samples. Bivariable and multivariable Poisson regression analyses offset by the natural logarithm of women's current age were performed to identify the socioeconomic and cultural factors that influenced fertility differentials in Morocco and Burundi. We first performed a bivariate Poisson regression analysis and all variables with a p-value < 0.2 in the bivariate analysis were considered in the multivariable Poisson regression model to assess the net effect of each of the selected socioeconomic and cultural factors. To facilitate the interpretation of the results, the coefficients were exponentiated to obtain the incidence rate ratio (IRR) [21,31]. In addition, the results were adjusted for marital status because marital status was strongly associated with the number of CEB. Variables with p<0.05 were reported as significantly associated with fertility differentials in both countries.

**Ethical considerations:** the protocols, consent forms, and data collection instruments for the 2003-04 Moroccan and 2010 Burundi DHS surveys were reviewed and approved by International Coaching Federation (ICF's) Institutional Review Board (IRB) and those of Morocco and Burundi, respectively. While ICF's IRB ensured that these surveys complied with the U.S. Department of Health and Human Services regulations for the protection of human subjects, the Morocco and Burundi IRBs ensured that the surveys complied with the laws and standards of each respective country [32]. In addition, informed oral consent was obtained from each respondent. For this study, the measure DHS program gave permission to access and download both data sets. We, on our part, agreed to treat the data from these two surveys as confidential [33].

## Results

**Figure 2:**
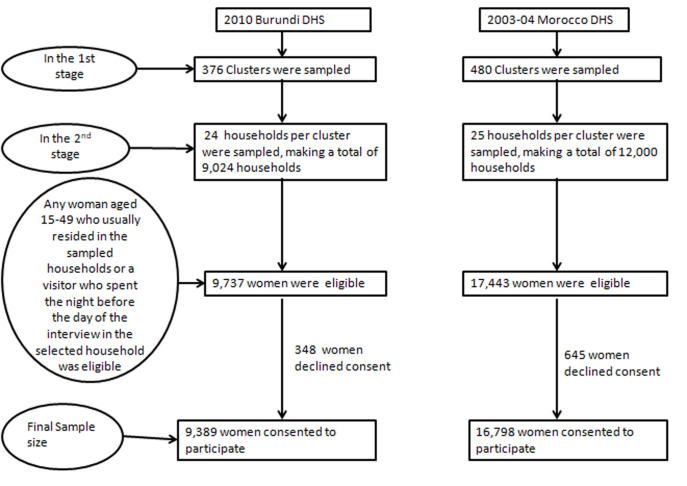
flow diagram showing recruitment of study participants and final sample size

**Participants:** in the first stage, 376 and 480 clusters were sampled in the 2010 Burundi and 2003-04 Morocco DHS surveys respectively using a systematic draw. Then, 9,024 (for Burundi) and 12,000 (for Morocco) households (24 and 25 households per cluster respectively) were sampled in the second stage using a systematic draw too. In these latter sampled households, respectively 9,737 and 17,443 women aged 15-49 were eligible, of whom 9,389 and 16,798 women consented to participate in the survey, representing participation rates of 96.4 and 96.3% respectively (Figure 2).

**Sociodemographic characteristics of the samples:** the sizes of the two samples were 9,389 and 16,798 women aged 15 - 49 years, with an average age of 27.72 (SD ± 9.62) and 29.62 (SD ± 9.99) for Burundi and Morocco respectively. The results showed that slightly more than three out of five women (61.7%) and slightly more than half of the women (52.9%) were less than 30 years of age for Burundi and Morocco respectively. For Burundi, most of the women (89.3%) lived in rural areas, while for Morocco most of the women (60.5%) lived in urban areas. Analysis of their marital status revealed that more than half of the women (57.7% in Burundi and 52.3% in Morocco) were married/living with a partner, while 33.2% and 42.1% in Burundi and Morocco respectively were still single. Regarding education level, 44.8% of the women were illiterate in Burundi compared to 50% in Morocco and only about 1% had a higher education level in Burundi compared to 5.3% in Morocco. Regarding their professional activity, our results showed that most of the women (77.6%) were working in an agricultural profession in Burundi while in Morocco most of the women (78.7%) were unemployed and only 2.7% were working in a modern profession in Burundi compared to 5.5% in Morocco. With respect to the household wealth index, the results indicate that 40.5% of women were from poor/very poor households in Burundi compared to 36.3% in Morocco (Table 1).

**Table 1:** sociodemographic characteristics of the samples

Variables categories		Burundi		Morocco	
		2010 DHS (N=9,389)		2003-04 DHS (N=16,798)	
		Frequency	Percentage	Frequency	Percentage
**Age groups (years)**					
	15-19	2,359	25.1	3,295	19.6
	20-24	1,832	19.5	3,011	17.9
	25-29	1,608	17.1	2,584	15.4
	30-34	1,064	11.3	2,245	13.4
	35-39	1,067	11.4	2,055	12.2
	40-44	745	7.9	1,921	11.4
	45-49	714	7.6	1,687	10.0
**Residence place**					
	Rural	8,387	89.3	6,639	39.5
	Urban	1,002	10.7	10,159	60.5
**Marital status**					
	Single	3,121	33.2	7,074	42.1
	Married/living with partner	5,421	57.7	8,782	52.3
	Divorced/separated	436	4.6	590	3.5
	widowed	411	4.4	352	2.1
**Women’s education**					
	No education	4,211	44.8	8,406	50.0
	Primary	4,042	43.1	3,317	19.7
	Secondary	1,054	11.2	4,189	24.9
	Higher	82	0.9	886	5.3
**Women’s occupation**:					
	Modern	254	2.7	926	5.5
	Agricultural	7,284	77.6	2,617	15.6
	Unemployed	1,827	19.5	13,220	78.7
	Other/don’t know	25	0.3	34	0.2
**Wealth quintile**					
	poorest	1898	20,2	2,959	17.6
	poorer	1910	20,3	3,145	18.7
	Middle	1854	19,7	3,380	20.1
	Richer	1811	19,3	3,600	21.4
	Richest	1916	20,4	3,714	22.1

DHS; demography health survey

**Socioeconomic and cultural factors that influenced fertility differentials in Burundi and Morocco during their fertility transition periods:** to identify the factors that influenced fertility differentials in Morocco and Burundi during their fertility transition periods, bivariable and multivariable Poisson regression analyses were performed.

**Bivariable analysis:** the total number of CEB ranged from 0 to 17 and from 0 to 16 children per woman with an average of 2.71 (SD ±2.89) and 1.88 (SD ±2.80) in Burundi and Morocco respectively. In the bivariable analysis factors like place of residence, marital status, women and husband's education and profession, wealth index, mass media access, the experience of infant mortality, age at first marriage, knowledge of any contraceptive methods, and modern contraceptive use were analyzed. The result of this analysis showed that all of the latter variables met the required condition (p-value < 0.2) to be considered in the multivariable Poisson regression analysis (Table 2).

**Table 2:** results of bivariable Poisson regression analysis of factors that influenced fertility differentials in Burundi and Morocco during their fertility transition periods

		Burundi		Morocco	
		2010 DHS (N=9,389)		2003-04 DHS (N=16,798)	
		UIRR (95% CI)	P value	UIRR (95%CI)	P value
**Residence place**					
	Urban (RC)	1.000		1.000	
	Rural	1.395 (1.276 - 1.525)	<0.001	1.450 (1.373 – 1.532)	<0.001
**Marital status**					;
	Single (RC)	1.000		1.000	
	Married/living together	29.819 (24.408 - 36.430)	<0.001	28.414 (27.189 - 29.015)	<0.001
	Divorced/separated	23.498 (19.163 - 28.813)	<0.001	11.147 (10.537 - 11.791)	<0.001
	Widowed	28.961 (23.609 - 35.527)	<0.001	26.863 (25.715 - 27.100)	<0.001
**Women's education**					
	Higher (RC)	1.000		1.000	
	secondary	0.992 (0.755 - 1.304)	0.954	1.193 (1.014 - 1.402)	0.033
	Primary	2.069 (1.602 - 2.671)	<0.001	1.969 (1.671 - 2.319)	<0.001
	No education	2.935 (2.278 - 3.783)	<0.001	3.722 (4.166 - 4.376)	<0.001
**Husband/partner's education**					
	Higher(RC)	1.000		1.000	
	secondary	1.402 (1.249 - 1.575)	<0.001	1.157 (1.094 - 1.223)	<0.001
	Primary	1.573 (1.422 - 1.739)	<0.001	1.479 (1.391 - 1.573)	<0.001
	No education	1.766 (1.599 - 1.951)	<0.001	1.848 (1.746 - 1.956)	<0.001
	Don’t know	0.123 (0.100 - 0.150)	<0.001	0.036 (0.028 - 0.047)	<0.001
**Women’s occupation:**					
	Modern (RC)	1.000		1.000	
	Agricultural	1.341 (1.205 - 1.493)	<0.001	1.536(1.381 - 1.708)	<0.001
	Unemployed	0.674 (0.581- 0.782)	<0.001	1.939 (1.778 – 2.114)	<0.001
	Others/don’t know	0.879 (0.640 - 1.208)	0.427	1.422(0.798 - 2.534)	0.231
**Husband/partner’s occupation**					
	Modern (RC)	1.000		1.000	
	Agricultural	1.270 (1.214 - 1.328)	<0.001	1.242(1.194 - 1.291)	<0.001
	Unemployed	1.256 (1.066 - 1.479)	<0.001	-	-
	Others/don’t know	0.070 (0.056 - 0.087)	<0.001	0.151(0.135 - 0.170)	<0.001
**Wealth quintile**					
	Richest (RC)	1.000		1.000	
	Richer	1.226 (1.148 - 1.309)	<0.001	1.106 (1.041 - 1.174)	0.001
	Middle	1.268 (1.188 - 1.355)	<0.001	1.331 (1.246 - 1.422)	<0.001
	Poorer	1.247 (1.170 - 1.330)	<0.001	1.632 (1.528 - 1.744)	<0.001
	Poorest	1.186 (1.109 - 1.268)	<0.001	1.809 (1.690 - 1.936)	<0.001
**Mass media access**					
	Yes (RC)	1.000		1.000	
	No	1.083 (1.036 - 1.133)	0.001	1.498 (1.412 - 1.588)	<0.001
**Experience of infant mortality**					
	No	1.000		1.000	
	Yes	2.460 (2.385 - 2.539)	<0.001	3.130 (3.030 - 3.234)	<0.001
**Age at first marriage**					
	≥ 20 years(RC)	1.000		1.000	
	16 - 19 years	1.226 (1.190 - 1.263)	<0.001	1.585 (1.536 - 1.635)	<0.001
	≤ 15 years	1.487 (1.426 - 1.550)	<0.001	1.908 (1.834 - 1.985)	<0.001
**Knowledge of any contraceptive methods**					
	Has knowledge (RC)	1.000		1.000	
	No knowledge	1.319 (1.234 - 1.434)	<0.001	1.687 (1.594 - 1.712)	<0.001
**Modern contraceptive use**					
	Yes (RC)	1.000		1.000	
	No	1.733 (1.707 - 1.759)	<0.001	1.653 (1.601 - 1.707)	<0.001

**Multivariable analysis:** after adjusting for women´s age and marital status, the following variables: place of residence, woman and husband's education and profession, wealth quintile, mass media access, experience of infant mortality, age at first marriage, knowledge of any contraceptive methods and modern contraceptive use remained significantly associated with fertility differentials in at least one of the two countries (Table 3). Indeed, women living in rural residences had 15.9% (AIRR = 1.159, 95% CI: 1.103 - 1.217, P=0.020) higher fertility rate compared to those living in urban residences in Burundi, while in Morocco the effect of residence place on fertility differentials was no longer significant. Our findings also revealed that illiterate women and those with only primary education had respectively higher fertility rates of 46.5% (AIRR=1.465, 95% CI: 1.241- 1.729, P <0.001) and 37.5% (AIRR=1.375, 95% CI: 1.168 - 1.619, P <0.001) in Burundi versus 42.8% (AIRR=1.428, 95% CI: 1.315 - 1.551, P <0.001) and 22.4% (AIRR=1.22.4,95% CI: 1.129 - 1.326, P <0.001) in Morocco when compared to those with higher education. Moreover, women whose husbands/partners are illiterate had a higher fertility rate of 24.2% (AIRR=1.242, 95% CI: 1.157 - 1.311, P = 0.006) in Burundi versus 14% (AIRR=1.140, 95% CI: 1.077 - 1.206, P <0.001) in Morocco when compared to those whose husbands/partners have higher education. Our findings also showed that women in agricultural occupations had a higher fertility rate of 33.2% (AIRR=1. 332, 95% CI: 1.263 - 1.401, P=0.004) in Burundi in reference to those in modern occupations. Similarly, women whose husbands/partners are in agricultural occupations had a higher fertility rate of 18% (AIRR=1.180, 95% CI: 1.034 - 1.228, P=0.001) in Burundi compared to those whose husbands/partners are in modern occupations. However, women's and husband's/partners´ occupations were not significant in the Moroccan context. Women living in the poorest and those in the poorer households had respectively higher fertility rates of 38.1% (AIRR= 1.381, 95% CI: 1.223 - 1.431, p<0.001) and 30.1% (AIRR=1.301, 95% CI: 1.211 - 1.387, p=0.003) in reference to those living in the richest households in Burundi, while in Morocco the effect of wealth quintile was not significant.

**Table 3:** results of multivariable Poisson regression analysis of factors that influenced fertility differentials in Burundi and Morocco during their fertility transition periods

Variables categories		Burundi		Morocco	
		2010 DHS (N=9,389)		2003-04 DHS (N=16,798)	
		AIRR (95% IC)	P-value	AIRR (95% IC)	P-value
**Residence lace**					
	Urban (RC)	1.000		1.000	
	Rural	1.159 (1.103 - 1.217)	0.020	1.031(0.989 - 1.074)	0.148
**Women’s education**					
	Higher (RC)	1.000		1.000	
	Secondary	1.220 (1.049 - 1.418)	<0.001	1.126 (1.042 - 1.218	0.003
	Primary	1.375 (1.168 - 1.619)	<0.001	1.224 (1.129 - 1.326)	<0.001
	No education	1.465 (1.241- 1.729)	<0.001	1.428 (1.315 - 1.551)	<0.001
**Husband/partner’s education**					
	Higher (RC)	1.000		1.000	
	Secondary	1.049 (0.921 - 1.194)	0.469	0.991(0.943 - 1.041)	0.719
	Primary	1.042 (0.906 - 1.197)	0.566	1.038 (0.978 - 1.101)	0.215
	No Education	1.242 (1.157 - 1.311)	0.006	1.140 (1.077 - 1.206)	<0.001
	Don’t know	1.136 (0.963 - 1.340)	0.131	1.043 (0.918 - 1.184)	0.519
**Women’s occupation**					
	Modern (RC)	1.000		1.000	
	Agricultural	1.332 (1.263 - 1.401)	0.004	1.106 (0.972 - 1.236)	0.058
	Unemployed	0.908 (0.834 - 0.989)	0.027	1.054 (0.965 - 1.116)	0.075
	Others/don’t know	0.819 (0.680 - 0.988)	0.037	0.991 (0.709 - 1.386)	0.959
**Husband/partner’s occupation**					
	Modern (RC)	1.000		1.000	
	Agricultural	1.180 (1.034 - 1.228)	0.001	1.110 (0.890 - 1.289)	0.123
	Unemployed	0.989 (0.859 - 1.139)	0.877	-	-
	Others/don’t know	1.036 (0.987 - 1.289)	0.069	0.930 (0.883 – 1.291)	0.765
**Wealth quintile**					
	Richest (RC)	1.000		1.000	
	Richer	0.923 (0.835 - 1.020)	0.072	0.968 (0.933 - 1.004)	0.077
	Middle	1.120 (1.022 - 1.203)	0.021	0.962 (0.916 - 1.010)	0.116
	Poorer	1.301 (1.211 - 1.387)	0.003	1.029 (0.972 - 1.090)	0.329
	Poorest	1.381 (1.223 - 1.431)	<0.001	1.028 (0.961 -1.098)	0.424
**Mass media access**					
	Yes(RC)	1.000		1.000	
	No	0.979 (0.952 - 1.007)	0.139	1.241(1.108 - 1.375)	0.006
**Experience of infant mortality**					
	No (RC)	1.000		1.000	
	Yes	1.602 (1.562 - 1.643)	<0.001	1.222 (1.184 - 1.361)	<0.001
**Age at first marriage**					
	≥ 20 years (RC)	1.000		1.000	
	16-19 years	1.155 (1.125 - 1.185)	<0.001	1.230 (1.119 - 1.378)	0.004
	≤ 15 years	1.313 (1.264 - 1.364)	<0.001	1.481(1.435 - 1.529)	<0.001
**Knowledge of any contraceptive methods**					
	Has knowledge (RC)	1.000		1.000	
	No knowledge	1.263 (1.125 - 1.310)	0.003	1.508 (1.409 - 1.613)	<0.001
**Modern contraceptive use**					
	Yes (RC)	1.000		1.000	
	No	1.520 (1.487 - 1.611)	<0.001	1.745 (1.627 - 1.863)	<0.001

**Note: AIRR**: Adjusted incident rate ratio; **RC**: Reference category; CI: confidence interval

Our findings also indicated that women who did not have access to mass media had a higher fertility rate of 24.1% (AIRR=1.241, 95% CI: 1.108 - 1.375, p=0.006) when compared to those who had access to mass media in Morocco, while in Burundi access to mass media was not significant. Women who already lost at least one child had a higher fertility rate of 60.2% (AIRR=1.602, 95% CI: 1.562 - 1.643, p<0.001) in Burundi versus 22.2% (AIRR=1.222, 95%CI: 1.184 - 1.361, p<0.001) in Morocco in reference to those who have not yet lost any child. Also, women who married at age 15 or before and those who married at age 16 - 19 had respectively higher fertility rates of 31.3% (AIRR=1.313, 95% CI: 1.264 - 1.364 p<0.001) and15.5 % (AIRR=1.155 95% CI: 1.125 - 1.185, p<0.001) in Burundi versus 48.1% (AIRR=1.481, 95% CI: 1.435 - 1.529, p<0.001) and 23 % (AIRR=1.230, 95% CI: 1.119 - 1.378, p=0.004) in Morocco when compared to those marrying at least at age 20. Women who did not have knowledge of any contraceptive method had a higher fertility rate of 26.3% (AIRR=1.263, 95% CI: 1.125 - 1.310, p=0.003) in Burundi versus 50.8% (AIRR= 1.508, 95% CI: 1.409 - 1.613, p<0.001) in Morocco as compared to those who had knowledge of any contraceptive method. Similarly, women who were not using modern contraceptive methods had a higher fertility rate of 52% (AIRR= 1.520, 95% CI: 1.487 - 1.611, p<0.001) in Burundi versus 74.5% (AIRR=1.745, 95% CI: 1.627 - 1.863, p<0.001) in Morocco when compared to those who were using modern contraceptive methods.

## Discussion

This study was conducted to first examine the factors that influenced fertility differentials in Morocco and Burundi during their fertility transition periods and then to compare the effects of these factors between the two countries. The total number of CEB varied from 0 to 17 and 0 to 16 per woman with an average of 2.71 (SD ±2.89) and 1.88 (SD ±2.80) in Burundi and Morocco respectively. Factors like rural residence, illiteracy or low level of education of women and their partners, the experience of infant mortality, early marriage, women and husband/partner´s agricultural profession, household poverty, lack of access to mass media, lack of knowledge of any contraceptive methods and failure to use modern contraceptive methods were found to be associated with high fertility (high number of CEB) in at least one of the two countries.

Indeed, our results revealed that rural residence was associated with high fertility in Burundi while its effect was no longer significant in Morocco. This finding could be attributed to the fact that Burundi remained the least urbanized country in the world with an urbanization rate of 10.6% in 2010 unlike Morocco whose urbanization rate was already 54.6% in 2004 [15]. Moreover, significant gaps in some widely known determinants of fertility differentials such as knowledge of any contraceptive method [21], and use of modern contraceptives [24] persist between urban and rural areas in Burundi. However, these inequalities were no longer significant between rural and urban areas in Morocco [3,17], demonstrating the convergence of childbearing behaviors between the two areas. The association between rural residence and high fertility was also reported in another previous study [34]. Similarly, a high level of education for men and especially for women was associated with a low fertility level in both countries. The role of women's higher education in lowering fertility was reported in several studies [3,17] However, the effect of women´s education was stronger in Burundi than in Morocco. This finding would be the result of the great success of the Moroccan national family planning program [3] in reducing socioeconomic disparities for access to FP services [35], unlike Burundi, where socioeconomic inequalities in access to FP services persist [23].

Women and husbands/partners´ agricultural activity was found to be associated with a high number of CEB in Burundi. Our results are consistent with those found in SSA [36] and in the Ivory Coast [37]. The effect of the agricultural profession was significant in Burundi, while it was not significant in Morocco. This observation may be due to the fact that Burundian agriculture remains traditional and therefore encourages most Burundian couples to give birth to many children given their important role in their parents' farming activities [21], unlike the Moroccan agricultural sector, which was already mechanized in 2004 [16] and therefore did not require a large workforce to induce Moroccan couples to give birth to many children as in Burundi. Household poverty was also found to be associated with a high number of CEB in Burundi. Our study finding is consistent with those of another study conducted in Nigeria [34] where the authors found that a higher mean of CEB was associated with household poverty and those of the study by Dribe *et al*. [27] who stated that the middle and wealthy classes experience a faster fertility transition compared to the poorer classes. However, the effect of the household wealth index was no longer significant in the Moroccan context based on our findings. This is probably due to the fact that in Morocco, all couples adopted a childbearing behavior in favor of a small family regardless of their socioeconomic status, especially through a poverty Malthusianism that would have influenced the fertility decline in rural areas, thus among poor households [35].

Similarly, access to mass media was significantly associated with low fertility in Morocco, while its effect was not significant in Burundi. This could be due to a lack of involvement of the Burundian mass media in spreading FP messages [21]. Access to mass media was also reported as a factor influencing the number of children desired as well as increased use of FP services in a study conducted in Bangladesh [30]. According to our findings, the experience of infant mortality was also associated with high fertility in both countries. Similar results were reported in previous studies [14,36] where the authors explain that in high infant mortality settings, the desired number of births may exceed the ideal number of children as a result of either a replacement effect after the death of a child or an insurance effect in anticipating such deaths, or both. Despite a significant improvement in child survival, the infant mortality rate remained higher in Burundi (96% in 2010) [6] compared to that of Morocco (47‰ in 2004) [18]. Our findings also showed that early marriage was associated with high fertility in both countries. Our study finding corroborates those of other studies that found that delay in first marriage was one of the main factors contributing to fertility differentials in Morocco [17] and Uganda [31].

While there was a substantial increase in the age at first marriage in Burundi [22] and Morocco [3], its effect was less important in Burundi than in Morocco. This finding could be attributed to the fact that marriage is the only recognized institution in which childbearing is permitted in Morocco [3,35], while in Burundi other illegal childbearing settings such as cohabitation [22] and unwanted teenage pregnancies [25] are still common. The evidence from our study also showed that the knowledge of any contraceptive methods and the use of modern contraceptives were associated with low fertility. Our findings are in agreement with those of other previous studies [3,34] which state that good knowledge of contraceptive methods and their use significantly influence fertility decline. Also, the effects of knowledge about any contraceptive method and the use of modern contraceptive methods were much greater in the Moroccan context than in Burundi. This finding would be the result of the great success of the national FP program in Morocco and the involvement of the private sector [3]. This has significantly contributed to increased awareness and contraceptive prevalence among Moroccan couples [3,17,38], unlike Burundi, where the unmet need for FP remains very high [23] and contraceptive prevalence very low [24].

Note that this study would be among the first to examine the heterogeneity of the effects of socioeconomic and cultural factors on fertility differentials during fertility transitions in countries with different socioeconomic and cultural backgrounds and with different fertility transition patterns. Moreover, the findings of this study can be generalized since the analysis is based on good quality and nationally representative data [6,18]. In terms of our study limitations, it should be noted that we were limited to examining the effects of socioeconomic and cultural factors that were available in the two DHS databases, as our study used secondary data analysis. Thus, other factors not identified by our study may have played a significant role in fertility differentials in at least one of these two countries.

## Conclusion

The results of this study revealed that both socioeconomic and cultural factors like rural residence, women´s illiteracy and agricultural profession, household poverty, infant mortality, early marriage, lack of knowledge of any contraceptives, and failure to use modern contraceptives were associated with a high number of children ever born in Burundi. However, in Morocco, socioeconomic factors like residence place, women´s agricultural profession, and household poverty were not significant. In this country, women´s illiteracy, lack of access to mass media, infant mortality, early marriage, lack of knowledge of any contraceptives, and failure to use modern contraceptives were associated with high fertility but with different effects than in Burundi. Intervention programs aimed at accelerating the fertility transition in Burundi and many other countries with slow fertility transitions should be designed and implemented according to the local context of each country.

### What is known about this topic

- *Many studies have focused on analyzing the factors that influenced fertility differentials in countries that have almost the same socioeconomic and cultural contexts;*
- *Many studies have focused on analyzing the factors that influenced fertility differentials in countries that have almost the same fertility transition pace;*
- *Few studies have examined the factors influencing fertility differentials and the variation in their effects in countries with different socioeconomic and cultural backgrounds and different fertility transition paces*.

### What this study adds

- *This study is one of the few studies that focus on the comparative analysis of socioeconomic and cultural factors that influenced fertility differentials during the fertility transition periods between two countries with very different socioeconomic and cultural backgrounds;*
- *This study may also be among the first ones to focus on the comparative analysis of variations in the effects of socioeconomic and cultural factors on fertility differentials during the fertility transition periods between two countries that have experienced a very different pace of fertility transition;*
- *The results of this study contribute to a better understanding of the extent to which Burundi and many other countries with very slow fertility transitions could accelerate them using or not the same interventions as those used by other countries, like Morocco, that succeeded in accelerating their fertility transitions*.
